# Evaluation of a Powered Stapler System with Gripping Surface Technology on Surgical Interventions Required During Laparoscopic Sleeve Gastrectomy

**DOI:** 10.1089/lap.2016.0513

**Published:** 2017-05-01

**Authors:** Elliott Fegelman, Susan Knippenberg, Michael Schwiers, Dimitrios Stefanidis, Keith S. Gersin, John D. Scott, Adolfo Z. Fernandez

**Affiliations:** ^1^ Ethicon, Inc., Cincinnati, Ohio.; ^2^ Division of Bariatric Surgery, Department of Surgery, Carolinas Healthcare System, Charlotte, North Carolina.; ^3^ Division of Bariatric and Metabolic Surgery, Greenville Health System, University of South Carolina School of Medicine—Greenville, Greenville, South Carolina.; ^4^ Department of General Surgery, Wake Forest University School of Medicine, Winston Salem, North Carolina.

**Keywords:** laparoscopic sleeve gastrectomy, powered stapler, staple line interventions

## Abstract

***Background:*** Transection of gastric tissue during laparoscopic sleeve gastrectomy (LSG) can be challenging. Reinforcing the staple line may decrease the incidence of issues requiring intervention.

***Methods:*** The objective of this study was to compare the number of intraoperative surgical interventions for a surgical stapler and reload system with Gripping Surface Technology (GST) to standard reloads in patients who underwent LSG. Patients who underwent elective LSG were enrolled. The study was conducted in two stages. For Stage 1, procedures were performed using a powered stapler and standard reloads. For Stage 2, a reload system with GST was used. The primary endpoint was surgical interventions for bleeding and/or staple line issues during transection of the greater curvature of the stomach. Propensity score matching was applied to create two groups similar in baseline characteristics and risk factors.

***Results:*** A total of 111 subjects were enrolled across four centers. Propensity-matched procedures were completed with the standard (*n* = 38) or GST reloads (*n* = 38). The mean number of interventions in the standard group was 1.9 (1.29) versus 1.1 (1.45) in the GST group. Nonparametric comparisons were statistically significant, indicating a reduction in the distribution of interventions for GST subjects (*P* = .0036 for matched pair data). Tissue slippage during transection was low for both groups. Intraoperative leak testing was negative in all procedures, and no procedures were converted to open.

***Conclusions:*** Use of the GST stapling system reduces the need for staple line interventions in LSG. Both stapling systems had an acceptable safety profile.

## Introduction

Laparoscopic sleeve gastrectomy (LSG) is a restrictive bariatric surgical procedure requiring resection of the stomach to restrict volume, resulting in weight loss and improved glucose homeostasis.^1^ Using a surgical stapler to transect tissue along the greater curvature of the stomach, volume is reduced by approximately 80%, leaving a “sleeve” that connects the esophagus to the small intestine. Relatively new, the first reported LSG procedure was performed in 1999.^2^ The procedure has seen growth in adoption over the past decade due to perceived ease of surgical technique, resolution of comorbidities, and excellent weight loss outcomes.^3^ During LSG, stapling thick gastric tissue can be challenging. For example, tissue may slip during firing, potentially adversely affecting staple line integrity.

Data have shown that LSG has a lower complication rate compared to Roux-en-Y gastric bypass.^4^ Acute complications associated with LSG include bleeding, abscess, and staple line leak.^5,6^ LSG and laparoscopic Roux-en-Y gastric bypass have similar reported leak rates.^7^ Staple line leaks represent the most dangerous and life threatening of these complications. A summary of 24 studies with 1749 patients reported a mean leak incidence of 2.7%.^8^ Better formed staples could potentially produce fewer leaks and bleeding complications postoperatively.^9–12^

Biological soft tissues are comprised of substantial amounts of interstitial fluid that “flow” in response to natural or applied pressures. The physics of surgical stapling requires tissue to be adequately coapted and free of motion during the stapling process so that a staple can be vertically lifted, accurately penetrate through the target tissue, and properly engage the anvil pocket to form into a proper B-shape. This is particularly challenging in thick gastric tissue as the surgeon must identify the target tissue's thickness, compressibility, and flow characteristics to adequately match staple leg length to the targeted tissue, facilitating optimal stapler performance and outcomes. Requirements for staple formation are linked to tissue compression; proper compression is essential to proper form.^13^ In addition, the increased access constraints of laparoscopy often require additional device features to improve access to the targeted tissue. For example, articulation mechanisms in laparoscopic staplers are sometimes needed to place the stapling end effector in the right orientation with respect to the targeted tissue for stapling.

We hypothesized that a newer generation stapling system, which accounts for tissue movements associated with the viscoelastic response of the tissue, would lead to fewer intraoperative staple line interventions (SLIs) compared to traditional stapling systems that lack this technology.

## Materials and Methods

This prospective, multicenter study (www.clinicaltrials.gov identifier NCT02358785) was executed from January to November 2015. There were four institutions, with two surgeons at each site. Following institutional review board approval at each participating site, patients scheduled for sleeve gastrectomy (SG) who provided informed consent were screened for participation. Key entry criteria included the following: meeting National Institute of Health (NIH) weight loss surgical criteria; body mass index (BMI) ≤60 kg/m^2^; ≥18 years; no previous gastrointestinal surgery; and no history of chronic steroid use.

In general, subjects were placed in a supine position on the operating table in reverse Trendelenburg position. The abdomen was insufflated and trocars placed. The greater curvature of the stomach was mobilized proximal to the pylorus. After mobilization of the stomach, a transoral bougie (size according to surgeon's discretion) was inserted into the pylorus and placed against the lesser curvature. Gastric transection began 3–6 cm proximal to the pylorus. The stapler was fired consecutively along the length of the bougie until the angle of His was reached. The entire staple line was inspected for integrity. The resected stomach was extracted through the periumbilical incision at the end of the procedure, followed by closure of the fascial defects.

The study was conducted in two stages. Procedures were performed according to each institution's standard-of-care using a powered stapler and standard reloads (ECHELON [ECH]) in Stage 1 and the ECHELON FLEX™ GST System (GST) in Stage 2. Two sites used staple line reinforcement (SLR) for all procedures.

Each surgeon completed all Stage 1 procedures before progression to Stage 2. Cartridges utilized for both groups were blue (closed staple height 1.5 mm), gold (1.8 mm), green (2.0 mm), and black (2.3 mm). Cartridge choice was at the discretion of the operating surgeon. The first procedure with the Gripping Surface Technology (GST) System was part of a learning curve and not included in the statistical analysis for interventions (the first study procedure with ECH was included in all analyses as all surgeons had previous experience with this device). All subjects were followed for approximately 4 weeks for safety and outcomes.

The primary study endpoint was SLIs required during transection of the greater curvature of the stomach. Interventions were defined as nonprophylactic actions taken in response to bleeding or other surgical issues along the staple line following tissue transection. Bleeding was classified as pulsatile or oozing lasting 15 seconds or more (requiring clip placement, oversewing, or targeted cautery). Staple lines (nonbleeding) requiring surgical intervention were defined as follows: (1) having air bubbles along the staple line that required oversewing; (2) any staple line irregularity requiring oversewing; or (3) other staple line issues requiring oversewing. Additional endpoints captured included demographics with medical/surgical history, procedure duration, cartridges used, usability of the device, procedure- and device-related complications, and surgeon satisfaction with the stapler used.

Data were collected using a centralized electronic data capture system and monitored throughout the study. Descriptive statistics, including number, mean, standard deviation (SD), median, minimum, and maximum, were calculated for all continuous variables; frequency and percentage were tabulated for all categorical variables. Propensity score matching was utilized to match subjects from Stage 1 (ECH) of the study to Stage 2 subjects (GST) for similar baseline characteristics/risk factors, including age, gender, race, BMI, waist circumference, and medical history of cardiometabolic conditions (diabetes, hypertension, and hypercholesterolemia). A sample size of at least 40 subjects per group was targeted to provide at least 80% power to demonstrate a 25% reduction in the number of interventions, assuming an average of six interventions per subject (PI estimate based on clinical practice). To facilitate matching and gain additional clinical experience, enrollment in the GST stage of the study was increased by 50%.

Within a matched pair, success was declared if the number of interventions in the GST subject was strictly less than for the ECH subject. The percentage of successes was compared to 0.5 using an exact binomial test. In addition, the distribution of the number of interventions was compared using Fisher's exact test. The median number of interventions was also compared using nonparametric methods. Comparisons were performed between the ECH and GST matched group, as well as between the ECH and GST all group. Safety was summarized through tabulation of adverse events. A significance level of .05 was used and nominal *P* values are reported. All statistical analyses were performed using SAS Version 9.3 (SAS Institute, Inc.).

## Results

A total of 111 subjects across four US centers were enrolled in the study. There were 38 procedures completed with standard cartridges (ECH) and 65 procedures completed with the GST system, of which 38 were matched to the ECH group. There were eight transition subjects (initial GST procedure by each surgeon). The study population included 19 (17.1%) men and 92 (82.9%) women with a mean (SD) age of 45.9 (11.61) years. BMI and waist circumference were generally well balanced across groups. Diabetes, hypertension, and hypercholesterolemia had a significantly higher incidence in the ECH group compared to the GST all group (*P* ≤ .05 for each comorbidity); however, none of the comparisons for ECH to the GST matched group was statistically significant (*P* ≥ .20 for each comorbidity) as comorbidity status was considered in the propensity score matching. A summary of baseline characteristics is provided in Table 1.

**Table 1.** Baseline Characteristics

*Variable*	*ECH (*N* = 38)*	*GST, matched (*N* = 38)*	*GST, all (*N* = 73)*	*Overall (*N* = 111)*
Age (years)				
Mean (SD)	47.8 (12.81)	46.7 (11.95)	44.9 (10.90)	45.9 (11.61)
Median (minimum, maximum)	46.5 (20, 69)	48.5 (21, 70)	44 (21, 70)	46 (20, 70)
Gender, *n* (%)				
Female	34 (89.5)	31 (81.6)	58 (79.5)	92 (82.9)
Race, *n* (%)				
Asian	0 (0.0)	0 (0.0)	1 (1.4)	1 (0.9)
Black or African American	12 (31.6)	10 (26.3)	17 (23.3)	29 (26.1)
Native Hawaiian or other Pacific Islander	0 (0.0)	0 (0.0)	1 (1.4)	1 (0.9)
White	26 (68.4)	28 (73.7)	54 (74.0)	80 (72.1)
Ethnicity, *n* (%)				
Hispanic	0 (0.0)	1 (2.6)	2 (2.7)	2 (1.8)
Non-Hispanic	38 (100.0)	37 (97.4)	71 (97.3)	109 (98.2)
BMI (kg/m^2^)				
Mean (SD)	44.0 (7.08)	42.5 (5.45)	43.1 (5.53)	43.4 (6.09)
Waist circumference, cm				
Mean (SD)	123.4 (15.63)	121.5 (16.96)	124.3 (18.4)	124.0 (17.41)
Diabetic, *n* (%)				
Yes	14 (36.8)	9 (23.7)	13 (17.8)	27 (24.3)
Hypertensive, *n* (%)				
Yes	27 (71.1)	23 (60.5)	34 (46.6)	61 (55.0)
Hypercholesterolemic, *n* (%)				
Yes	20 (52.6)	14 (36.8)	18 (24.7)	38 (34.2)

BMI, body mass index; ECH, ECHELON; GST, Gripping Surface Technology; SD, standard deviation.

Mean operating time was 74.3 (± 23.1) minutes for ECH and 68.0 (± 23.3) minutes for GST; no procedures were converted to open and blood loss was minimal. Time to complete transection of the stomach was 15.1 (± 5.0) minutes for ECH and 13.1 (± 4.3) minutes for GST. Intraoperative leak testing was negative for all 57 procedures in which it was performed.

The number of SLIs in the ECH group (*N* = 38) was 1.9 ± 1.29, with a maximum of four interventions for any procedure (Table 2). The matched GST group had a mean of 1.1 ± 1.45 interventions. This increased to 1.3 ± 1.84 for all GST subjects.

**Table 2.** Staple Line Interventions

*Variable*	*ECH (*N* = 38)*	*GST, matched (*N* = 38)*	*GST, all (*N* = 65)*
Staple line interventions			
Mean (SD)	1.9 (1.29)	1.1 (1.45)	1.3 (1.84)
Median (minimum, maximum)	2 (0, 4)	1 (0, 7)	1 (0,9)
95% Exact CI	1.5, 2.3	0.6, 1.5	0.9, 1.8
Intervention distribution, *n* (%)			
0	7 (18)	17 (45)	27 (42)
1	7 (18)	11 (29)	19 (29)
2	12 (32)	7 (18)	10 (15)
3	7 (18)	0 (0)	1 (2)
4	5 (13)	2 (5)	5 (8)
7	0 (0)	1 (3)	2 (3)
9	0 (0)	0 (0)	1 (2)

Interventions for bleeding or other surgical issues along the staple line following tissue transection.

CI, confidence interval; ECH, ECHELON; GST, Gripping Surface Technology; SD, standard deviation.

**FIG. 1.**
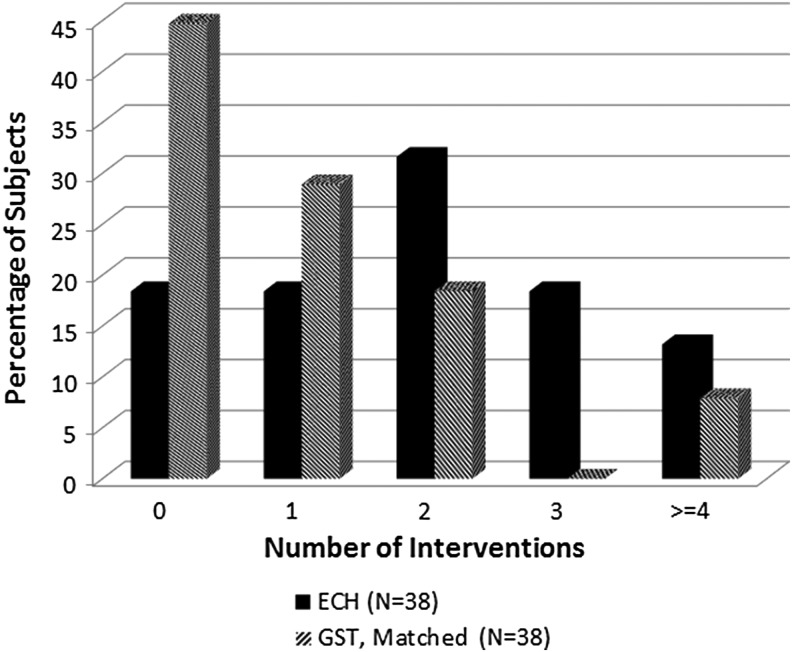
Interventions for matched pairs as a percentage of subjects who had their procedures performed with ECH cartridges or GST. GST, Gripping Surface Technology.

As illustrated in Figure 1, Stage 2 (GST) matched pairs had fewer interventions than Stage 1 (ECH). The maximum number of interventions in any procedure was nine (GST group). Endoclips were the most frequently used intervention (> 90%) for pulsatile bleeding, while cautery was used most often for oozing (> 45%). Oversewing was performed for bleeding in nine procedures and for irregular staple lines in three procedures.

The comparison of the distribution of number of interventions between groups was statistically significant (*P* = .0036). A nonparametric comparison of the median number of interventions between groups was also significant (*P* = .0018). Only three subjects (7.9%) in the GST matched group required three or more interventions compared to 12 ECH subjects (31.6%).

Interventions by cartridge relative to the total number of firings were also reported. Blue cartridges were utilized for transection most frequently (> 40% across groups). Although black cartridges were used for fewer firings than other cartridges, interventions were required more frequently, with 53% of black cartridge firings requiring an intervention in the ECH group and 44% of black cartridges requiring an intervention in the GST matched group (42% GST all). Up to seven cartridges were used per procedure. Cartridge utilization was similar between the ECH and GST matched groups. Blue cartridges were used in 42.2% of ECH firings and 41.6% of GST matched firings. Similarly, gold cartridges were used in 19.1% and 19.8% of firings for ECH and GST matched groups, respectively, Green cartridges in 23.0% and 22.3%, and black cartridges were used in 15.7% of ECH firings and 16.2% of GST matched firings. Surgeons typically initiated transection of the stomach with the black or green cartridge and completed with the blue cartridge at the angle of His. The most frequent pattern (16 occurrences) used under this protocol was green, blue, blue, blue, and blue.

**FIG. 2.**
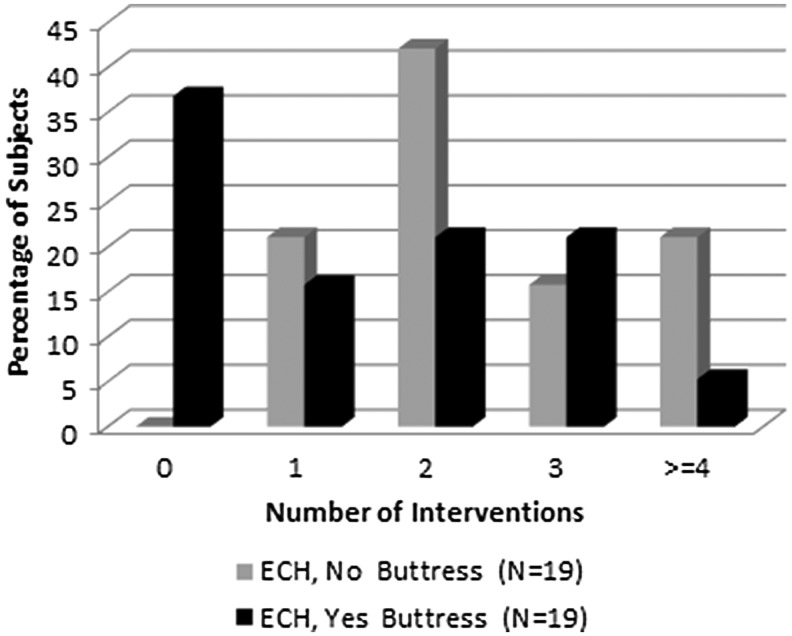
Interventions by use of staple line reinforcement in Stage 1.

**FIG. 3.**
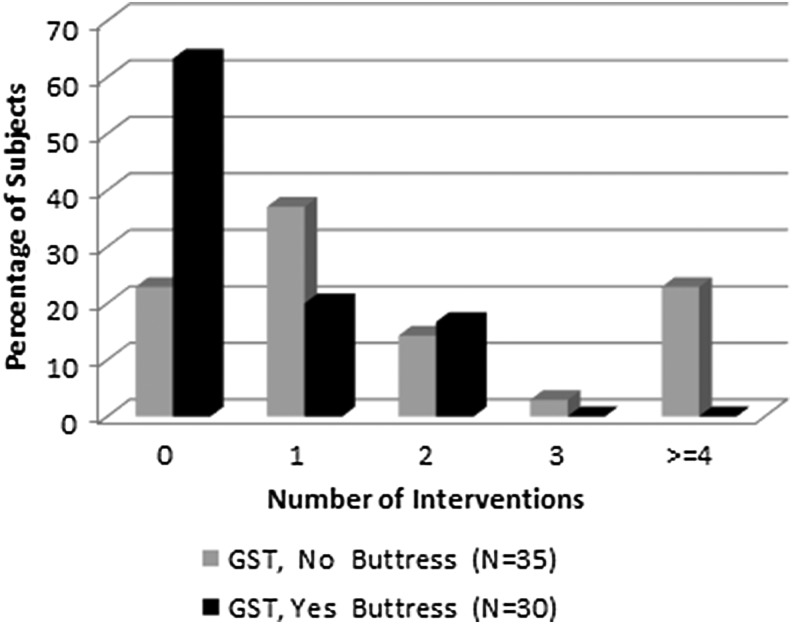
Interventions by use of staple line reinforcement in Stage 2.

As detailed in Table 3, two of the four study sites prophylactically used SLR across procedures (19 ECH, 30 GST). All procedures with ECH required at least one intervention when no SLR was used (Fig. 2). With GST, eight subjects without an SLR (22.9%) and 19 subjects with an SLR (63.3%) did not require an intervention (Fig. 3). When SLR was used with GST, no subjects required more than two interventions.

**Table 3.** Use of Staple Line Reinforcement

*Variable*	*ECH (*N* = 38)*	*GST, all (*N* = 65)*
Interventions, subjects without prophylactic use of SLR		
*n* (%)	19 (50)	35 (54)
Mean (SD)	2.4 (1.07)	2.0 (2.20)
Median (minimum, maximum)	2 (1, 4)	1 (0, 9)
Intervention distribution, *n* (%)		
0	—	8 (22.9)
1	4 (21.1)	13 (37.1)
2	8 (42.1)	5 (14.3)
3	3 (15.8)	1 (2.9)
≥4	4 (21.1)	8 (22.9)
Interventions, subjects with prophylactic use of SLR		
*n* (%)	19 (50)	30 (46)
Mean (SD)	1.4 (1.35)	0.5 (0.78)
Median (minimum, maximum)	1 (0, 4)	0 (0, 2)
Intervention distribution, *n* (%)		
0	7 (36.8)	19 (63.3)
1	3 (15.8)	6 (20.0)
2	4 (21.1)	5 (16.7)
3	4 (21.1)	—
≥4	1 (5.3)	—

ECH, ECHELON; GST, Gripping Surface Technology; SD, standard deviation; SLR, staple line reinforcement.

Overall, surgeons were satisfied with the performance and usability of the device across groups. Tissue slippage during transection was low for both the ECH and GST groups. In the full analysis set for the GST group, only three of the 337 firings (0.99%) had any tissue slippage. There was one occurrence of extensive slippage across all subjects (ECH). All surgeons who used articulation reported that it made the procedure easier to perform. Of these, the angle range was sufficient in all but two procedures (one ECH and one GST).

All enrolled subjects (*N* = 111) were followed for safety. Common adverse events (frequency >5%), with a possible relationship to the procedure or study device, are summarized in Table 4. A total of 120 events were reported in 62 (55.9%) subjects. All events were anticipated, and there were no deaths reported during the study. No occurrences of leak at the staple line were reported. The most common events reported were nausea, diarrhea, and constipation, consistent with the anticipated postoperative safety profile for this procedure.

**Table 4.** Adverse Event Summary

*Adverse event (subjects)*	*ECH (*N* = 38)*	*GST, all (*N* = 73)*	*Overall (*N* = 111)*
Nausea, *n* (%)	10 (26)	21 (29)	31 (28)
Diarrhea, *n* (%)	5 (13)	13 (18)	18 (16)
Constipation, *n* (%)	4 (11)	9 (12)	13 (12)
Vomiting, *n* (%)	4 (11)	5 (7)	9 (8)
Abdominal pain, *n* (%)	2 (5)	1 (1)	3 (3)
Dehydration, *n* (%)	2 (5)	2 (3)	4 (4)

ECH, ECHELON; GST, Gripping Surface Technology.

## Discussion

In general, even when optimal surgical techniques are used, significant compression may still be needed when transecting thick tissue. It is clear that the resulting movement, either from the device or tissue, during this phase of firing can impact staple form. This threat of staple line disruption is the greatest concern for surgeons performing LSG, although causes for disruption are not well known. Recognizing that a secure staple line is critical to a successful procedural outcome, we embarked on a research program to develop a system that would minimize tissue slippage and increase staple line security.

It is recognized that the assessment of staple line integrity is most optimally assessed through the evaluation of leaks. However, the objective of this study was to investigate whether changes to the gripping surface of the cartridge deck could have an impact on the frequency of interventions, a potential surrogate for staple line disruption, thus providing information for whether this technology could affect leak rates in future studies. We evaluated the number of interventions during each surgery as a surrogate for leaks with the understanding that poorly formed staple lines or excessive bleeding could represent inadequate staple lines.

In this study, use of the GST stapling system reduced the need for staple line interventions during LSG. Approximately 74% of the GST matched subjects had 0 or 1 interventions, while approximately 36% of the procedures with ECH had 0 or 1 interventions. Two sites used SLR for all procedures in both stages, and thus, any confounding would be distributed among both stages. Furthermore, while the analysis by SLR use overall showed fewer interventions, the data were still favorable toward GST for a reduction in the number of interventions. Surgeons were generally satisfied with the GST system and reported that articulation of the stapler made the procedure easier to perform.

Limitations of the study included a small sample size, lack of randomization, and low overall number of staple line interventions per procedure.

## Conclusions

Use of the GST stapling system reduces the need for staple line interventions in LSG. While the use of SLR may have an impact on the need for surgical interventions, the small sample size in this study suggests that additional research is needed. No unexpected safety issues were observed in this initial clinical trial, suggesting that the GST system has an acceptable safety profile in LSG procedures.
